# Cat-Eye Syndrome: A Report of Two Cases and Literature Review

**DOI:** 10.7759/cureus.26316

**Published:** 2022-06-25

**Authors:** Nélia S Gaspar, Gustavo Rocha, Ana Grangeia, Henrique C Soares

**Affiliations:** 1 Pediatrics, Centro Hospitalar do Médio Tejo, Torres Novas, PRT; 2 Neonatology, Centro Hospitalar Universitário de São João, Porto, PRT; 3 Genetics, Centro Hospitalar Universitário de São João, Porto, PRT

**Keywords:** congenital heart disease, craniofacial anomalies, cat eye syndrome, ears anomalies, anal atresia

## Abstract

Cat-eye syndrome is a rare genetic disease that involves the proximal long (q) arm of chromosome 22. The classic clinical triad includes coloboma of the iris, ears, and anal malformations. This syndrome was named “cat eye” due to the vertical coloboma of the iris. However, the spectrum of clinical manifestations is variable, and the iris coloboma may be absent in 40-50% of cases. Association with congenital heart disease is also frequent and its diagnosis should raise suspicion of a genetic condition. We describe two cases of male infants affected by the cat-eye syndrome, of which no one presented the classic clinical triad. One of them had unpredictable complications that led to prolonged neonatal intensive care unit stay. Although having distinct phenotypes, the diagnosis in both cases was made through nonobstructive total anomalous pulmonary venous return, anal imperforation, and craniofacial anomalies. Iris coloboma was an important clue only in one of them. Prenatal diagnosis is a challenge, such that a genetic study is essential for a final diagnosis in the absence of the classic triad.

## Introduction

Cat-eye syndrome (CES), also known as Schmid-Fraccaro syndrome or syndrome of partial tetrasomy of chromosome 22, is a rare genetic disease affecting one in 150,000 people [1]. It is caused by duplication or triplication of the proximal long (q) arm of chromosome 22, due to a small supernumerary marker chromosome (sSMC) [1-3]. The proximal region of the (q) arm of chromosome 22 is associated with genomic disorders with intellectual disabilities and congenital malformations, such as CES and DiGeorge syndrome - the latter being the most common genomic disorder on 22q11, affecting one in 4,000 live births [4].

The proximal region of the (q) arm of chromosome 22 includes genes responsible for typical ophthalmologic findings [1]. “Cat eye” stems from the vertical coloboma of the iris, conferring the “cat eye” characteristic phenotype, and is present in some patients [1,5]. In 1965, the first report on sSMC in a child with polymalformative syndrome with coloboma was linked to other congenital anomalies [5].

The classic clinical presentation triad of CES is characterized by coloboma of the iris, ear, and anal malformations [1]. However, the spectrum of malformations is widely variable [1-4]. The presence of congenital heart disease is frequent, with total anomalous pulmonary venous return (TAPVR) as one of the most common [4]. Cat-eye syndrome phenotype includes preauricular pits or tags, down-slanting palpebral fissures, hypertelorism, and renal malformations [3]. Moreover, almost half the patients have mild or moderate developmental delay, but growth is usually normal [1,3]. We report two cases of CES, with different phenotypical characteristics and favorable prognoses.

This article was previously presented as a meeting abstract at the 21st Congresso Nacional de Pediatria, Portugal, on October 27, 2021.

## Case presentation

Case 1

**Figure 1:**
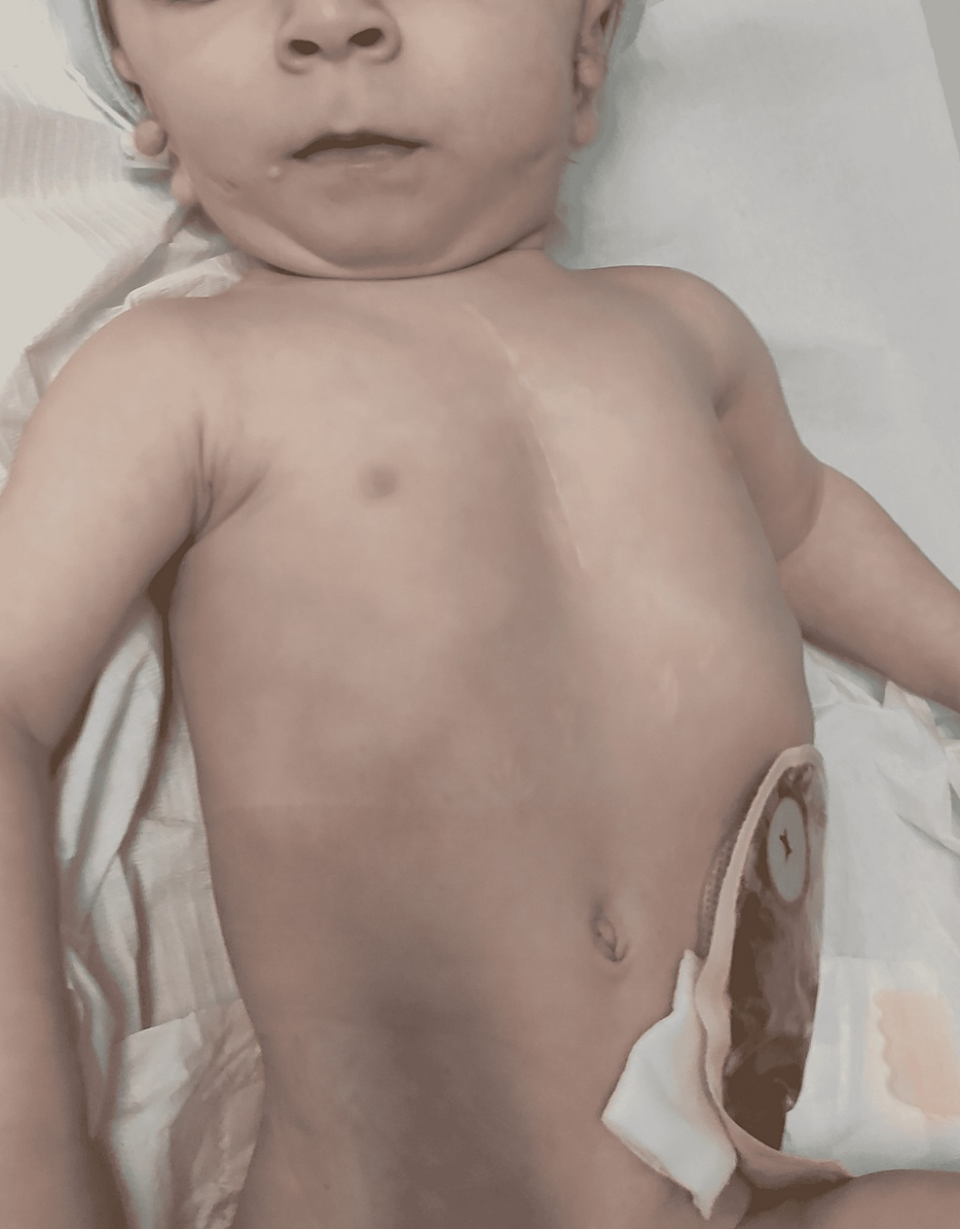
Patient in case 1 is using hearing aids and waiting for colostomy closure.

A late preterm male infant was delivered by cesarean section at 36 weeks and six days of gestation, to a 1G/0P, 32-year-old mother. The pregnancy was supervised with oligohydramnios detected on the ultrasound scan in the third trimester, without any complications. Apgar scores were 9, 10, and 10 at the first, fifth, and 10th minutes, respectively. The parents were non-consanguineous and healthy. After birth, the initial examination revealed an adequate for gestational age infant with a birth weight of 2,490 grams, with craniofacial dysmorphism, including hemifacial microsomia with microtia and agenesis of the external auditory meatus on the right side, bilateral preauricular pits and tags, pinpoint openings in the mandibular region, suggestive of branchial cleft sinus tract, ocular hypertelorism, and anal atresia. Additionally, a systolic heart murmur was detected. He was admitted to the neonatal intensive care unit (NICU) due to cyanosis and suspected congenital heart disease. After cardiac evaluation, a diagnosis of nonobstructive TAPVR was established, with pulmonary veins draining into the right atrium, near the superior vena cava entrance, with dilated right cavities and small left cavities. He was submitted to a sigmoid colostomy with a mucous fistula on day four of his life and TAPVR correction on day 24, both with favorable outcomes. The ophthalmologic evaluation excluded the presence of coloboma, and the otorhinolaryngologic evaluation diagnosed moderate hearing loss on the right and severe loss on the left. Genetic evaluation by karyotyping of a peripheral blood sample revealed an additional dicentric sSMC (47, XY, +idic {22} {q11.21}) and an array of comparative genome hybridization (aCGH) showed a CES chromosome (arr22q11.1q11.21{17,068,189_18,894,894} x4), confirming a diagnosis of CES. He is currently one year old and is followed by a multidisciplinary team using a bone conduction prosthesis and waiting for an auditory osseointegrated implant and colostomy closure (Figure 1).

Case 2

A term neonate was vaginally delivered at 38 weeks and four days of gestation, to a 1G/0P, 27-year-old mother. Apgar scores were 9, 10, and 10 at the first, fifth, and 10th minutes, respectively. The pregnancy was supervised, with a prenatal diagnosis of intestinal loop distension, but no other complications. The parents were non-consanguineous. After birth, the initial examination revealed an adequate for gestational age male infant with a birth weight of 3,480 grams, with anal atresia and hypoplasia of the left preauricular region, as to why he was admitted to the NICU. After cardiac evaluation, a TAPVR was diagnosed (with pulmonary veins draining into the right innominate vein) and ophthalmologic observation identified a right unilateral iris coloboma. Additionally, a recto-urethral fistula and left hydronephrosis were diagnosed. He underwent a sigmoid colostomy on day three and a TAPVR surgical correction on day 26 of life. During the postoperative period of cardiothoracic surgery, he developed a bilateral chylothorax, right pneumothorax, *Staphylococcus epidermidis* sepsis, and deprivation syndrome, which led to a prolonged NICU stay of 52 days. The genetic study revealed a partial tetrasomy of chromosome 22. At 23 months, he was submitted for colostomy closure, with a favorable outcome. Currently, he is five years old, with a mild global developmental delay, recurrent pneumonia, and intermittent fecal incontinence.

## Discussion

We report two confirmed neonatal cases of CES. In both cases, the obstetrical history underlined nonspecific fetal ultrasound anomalies (oligohydramnios and intestinal loop distension - in cases 1 and 2, respectively), with a malformation syndrome detected at birth, requiring admission to the NICU.

Cat-eye syndrome stems from trisomy or tetrasomy of the proximal long (q) arm of chromosome 22, which derives from sSMC [1-3]. The diagnosis of sSMC may require molecular and cytogenetic studies, including chromosomal analysis by karyotyping, fluorescent in situ hybridization (FISH), and array CGH [3,4-7]. Prenatal diagnosis is always a challenge since the fetal ultrasound findings are unspecific, but an sSMC of chromosome 22 detected at amniocentesis should alert CES [7-9]. In both cases, CES was never suspected before birth, and amniocentesis was not performed.

The clinical presentation is variable and does not correlate with genetic content [1,3,6-11]. The classic clinical presentation triad is coloboma of iris, ears and anal malformations, found in around 40% of newborns [1]. Phenotypical characteristics are widespread, demonstrated by the fact that coloboma, which confers the typical appearance of “cat eye” to these patients, and are present in only 50-60% of cases [1,3]. Coloboma was found only in case 2 and was the main sign of clinical suspicion. Also, none of our patients meets the classic clinical presentation; however, anal malformation, one of the most frequent CES characteristics, which is present in about 80% of patients, was diagnosed in both [3].

Although congenital heart anomalies do not belong to the clinical presentation triad, they are present in about 60% of affected patients, with atrioventricular septal defect and TAPVR being the most frequently identified [3]. Total anomalous pulmonary venous return is a rare congenital heart defect and its diagnosis by transthoracic echocardiogram should raise suspicion of a genetic defect. In a case series study of 71 patients with CES, TAPVR was found in 21% [2]. Total anomalous pulmonary venous return was diagnosed in both cases and the surgical correction was necessary during the NICU stay. In addition to the syndrome denomination, one of the most frequent clinical findings is preauricular pits/tags, which occur in 85% of patients and present only in case 1 [8]. 

Major features of CES include urogenital malformations in 70%, such as abnormal male external genitalia, and renal agenesis, although none was observed in the described cases [3,7]. The minor anomalies of CES include craniofacial dysmorphism, such as downslanting palpebral fissures, hypertelorism, strabismus, epicanthic folds, microphthalmia, low-set ears, and micrognathia [3,6]. Mental development may also be affected, with mild/moderate or severe intellectual disability (almost 50% and 7% of the cases, respectively) [3].

Treatment depends on diagnosed anomalies and usually includes supportive care with surgical corrections. The global prognosis is favorable, including for those with intellectual disability [1,8]. However, early detection of malformations and therapeutic guidance is essential, especially regarding congenital heart disease and anorectal malformation, which may require surgical treatment [1,8].

Table 1 summarizes anomalies found in reported cases and compares them with those described in the literature [1-3,6,7,11]. Although none of the described cases has innovative features, they illustrate two distinct phenotypes of the same syndrome with different clinical courses. Although the overall prognosis is historically favorable, patients may have prolonged NICU stay, which has a substantial impact on morbidity.

**Table 1:** Comparison of clinical anomalies in the cases reported here and in those described in literature.

Clinical findings described in literature	Prevalence (%)	Case 1	Case 2
Iris coloboma	50-60	-	+
Craniofacial anomalies			
Micrognathia		+	-
Hemifacial hypoplasia		+	+
Hypertelorism		+	-
Preauricular tags or pits	85	+	-
Downslanting palpebral fissures		+	-
Strabismus		-	-
Epicanthic folds		-	-
Low-set ears		-	-
Ear malformation			
Microtia		+	-
External auditory meatus agenesis		+	-
Anal malformation	80		
Anal imperforation		+	+
Rectal fistula		-	+
Congenital heart disease	60		
Anomalous pulmonary venous return		+	+
Fallot tetralogy		-	-
Atrioventricular septal defect		-	-
Urogenital malformation	70		
Abnormal male external genitalia		-	-
Renal agenesis		-	-
Hydronephrosis		-	+
Intellectual disability	50	-	+

## Conclusions

In conclusion, CES is a rare genetic disease with a difficult prenatal diagnosis. Its denomination derives from typical ophthalmological findings; however, it may be absent in almost half the cases. Total anomalous pulmonary venous return is a hallmark feature and its diagnosis should raise the suspicion of a genetic syndrome. A genetic study may be crucial in the absence of the classic clinical triad relating to the syndrome. The authors stress the need for a high level of suspicion in cases without the “cat eye.”
